# Don’t face writer’s block alone

**DOI:** 10.7554/eLife.92931

**Published:** 2023-09-26

**Authors:** Bruna Martins Garcia

**Affiliations:** 1 Max Planck Institute for Biology of Ageing Cologne Germany

**Keywords:** sparks of change, research culture, writing, writing club, career, early-career researchers

## Abstract

Creating a writing club allowed a Brazilian PhD student to confront her fears, improve her English and, ultimately, change the way she sees research.

As female researcher hailing from Brazil, I always thought that I’d need to show self-sufficiency to be taken seriously in science. I believed that the most important thing about my PhD would be to prove that I was ready to be the ‘independent researcher’ described in so many ads for postdoctoral positions. To me, this meant I had to demonstrate that I could overcome any issue on my own. So, when I started to find it difficult to express myself in writing, my response was also to try and fix the problem alone.

It all came to a head when I moved to Germany for my PhD and was forced to communicate in English, a language I hadn’t had the chance to learn properly. I managed to improve my vocabulary and grammar in a few months, but I quickly realised that this wouldn’t be enough. There are many differences between writing in Portuguese (the national language of Brazil) and writing in English, including the ways that ideas are organized, and arguments are structured. In particular, Brazilians are not afraid to use lots of redundant adjectives: however, when this is done in English, a piece of writing is likely to be criticized for being too long, too confusing or both.

I started to become self-conscious about expressing myself, in particular in writing. In my mind, mistakes in oral communication were transient and therefore forgivable; mistakes in written communications, on the other hand, were much more serious. There was something familiar about these feelings. Back in Brazil an 11-year-old me had received a fellowship to study at a private school, which was when I first realized that some pupils from other families were much more sophisticated in their use of Portuguese than I was. I think my initial decision to study engineering stemmed in part from wanting to focus on maths and other scientific topics that relied less on language skills.

As I progressed through my PhD, however, I had to write more and more reports, as well as applications for grants and fellowships, and my drafts kept coming back full of corrections and comments. As my belief in my ability to convey my thoughts in writing deteriorated, I was too ashamed to share my struggles with my peers, even though drafting something as simple as an e-mail had often become torturous for me. Indeed, I started to avoid writing as much as possible, and was genuinely worried that my problems with writing would stop me from becoming a good scientist. I know I missed out on several job opportunities and conferences, for example, simply because I couldn’t bring myself to put together a cover letter or an abstract.

I tried hard to improve, but I felt I was making little progress. I was told that I needed to practise, but simply writing something without knowing why I was writing it seemed like a waste of time. I would sometimes ask a lab mate for feedback, but quickly started to feel that I was bothering him. I also surrounded myself with manuals about scientific writing but found it difficult to keep up with the homework on my own.

After a while, I realised that I needed another approach. I began to toy with the idea of starting a writing club, something that had been suggested in two of my books. At first, I was skeptical. Writing had always seemed like a solo activity to me, and it was scary to think that I could be revealing my fears to people who wouldn’t be able to understand them. But after thinking for a few days, I took the plunge; I texted four colleagues who I thought may like the idea and they all agreed right away. With this, our writing club was born.

On the surface, we didn’t seem to have much in common: only one of us was a native English speaker, and we were all from different cultures, research fields and PhD levels. Yet, the first time we came together, we realized that we had been facing similar struggles. We were all trying to deal with these difficulties alone but had become demotivated and reluctant to write as a result. Having my thoughts and feelings validated by the group was a great source of comfort, and it was really motivating to see how keen we were to improve together.

For about 10 months we met every week to go over two books on scientific writing, give each other feedback on the exercises they proposed, and share other relevant materials (see Box 1). We also started to bring our own writings to the club. The group quickly became a judgment-free zone where we could find reliable feedback on our papers, theses, proposals – even this article. In this close and trusted community, it was easier to face my fears. Little by little, the shame of coming up with a bad sentence was replaced by wanting to know how the others thought it could be improved. Being responsible for our weekly study plan and mediating our discussions also meant that I couldn’t avoid writing-related activities anymore; in fact, it motivated me to put even more effort into them. In truth, I was surprised by how much the group was making something that I hated easier. We could all see quick progress, and I began to receive compliments from my peers and PI. Thanks to the club, writing stopped being torture for us; we’ve even started to like it!

Box 1. **Six tips to start a writing club.**

- **Have the right supporting materials:** We used the books “Writing Science: How to Write Papers That Get Cited and Proposals That Get Funded” and “The Scientist’s Guide to Writing”. We had a week to read one chapter and do the related exercises before the next meeting.
- **Find a discussion lead:** The role of the discussion lead is to guide the group through the main points raised in the chapter studied that week, and to relay the correct answers for the related exercises. The lead then coordinates the resulting discussion, ensuring that every member has the opportunity to express their thoughts.
- **Recruit the right people:** Five or six members is a good number to start with. Bringing together individuals from different nationalities, cultures and scientific backgrounds enriches the discussions and, in our experience, gave us the opportunity to practice communicating science to a diverse audience. Ideally, the group should include at least one native English speaker too.
- **Create a judgment-free environment:** This is the most important element. Members should feel safe to share imperfect work. They need to know that they can make mistakes without feeling ashamed or embarrassed.
- **Share your own texts:** Working on your own materials enhances motivation as you're familiar with the content and can immediately reap the benefits. For instance, you can collaborate with others to improve an old abstract and share it with your PI to gauge their feedback.
- **Have fun!:** Fun will keep you motivated. We regularly brought sweets and tea to our meetings and shared videos and memes about scientific writing between sessions. You can also plan social events together.

What I couldn’t foresee at the time was that this experience would help me let go of my individualistic beliefs. I’ve realised that being an independent scientist doesn’t mean never needing support; instead, it’s about knowing how to actively find help and figuring out who can provide it. I don’t feel that I have to solve every problem alone anymore. As I advance in my career, I now seek like-minded colleagues who face similar challenges so we can tackle them together. I also hope other PhD students realize the strength in facing challenges collectively, rather than trying to prove themselves alone. Accepting help doesn’t make you less of a scientist – just a better one.

## Share your experiences

This article is a Sparks of Change column, where people around the world share moments that illustrate how research culture is or should be changing. Have an interesting story to tell? See what we’re looking for and the best ways to get in touch here.

